# Vascular Endothelial Growth Factor Induces CXCL1 Chemokine Release via JNK and PI-3K-Dependent Pathways in Human Lung Carcinoma Epithelial Cells

**DOI:** 10.3390/ijms140510090

**Published:** 2013-05-10

**Authors:** Huey-Ming Lo, Jiunn-Min Shieh, Chih-Li Chen, Chih-Jen Tsou, Wen-Bin Wu

**Affiliations:** 1 School of Medicine, Fu-Jen Catholic University, New Taipei City 24205, Taiwan; E-Mails: M006459@ms.skh.org.tw (H.-M.L.); lins2023@mail.fju.edu.tw (C.-L.C.); sakura29300921@hotmail.com (C.-J.T.); 2 Section of Cardiology, Department of Internal Medicine, Shin Kong Wu Ho-Su Memorial Hospital, Taipei 11101, Taiwan; 3 Department of Internal Medicine, Chi-Mei Medical Center, Tainan 71004, Taiwan; E-Mail: shieh225@ms29.hinet.net

**Keywords:** A549, chemokine, CXCL1, GRO alpha, signaling, VEGF

## Abstract

Lung cancer cells express different chemokines and chemokine receptors that modulate leukocyte infiltration within tumor microenvironment. In this study we screened several mediators/growth factors on CXCL1 release in human carcinoma epithelial cells. Of the tested mediators, VEGF was found to have a robust increase in causing CXCL1 release. VEGF stimulated CXCL1 release and mRNA expression in a time- and concentration-dependent manner. The release was inhibited by the VEGF receptor antagonists and the JNK, PI-3K, tyrosine kinase, and transcription inhibitors. In parallel, VEGF induced JNK, PI3K and Akt activation. Strikingly, among these inhibitors only the JNK inhibitor could reduce VEGF-induced CXCL1 mRNA expression, suggesting that JNK participated in VEGF-induced CXCL1 synthesis, whereas PI-3K was responsible for cellular CXCL1 secretory process. In addition, the steroid dexamethasone and TGF-β suppressed CXCL1 release through a transcriptional regulation. We also showed that cells stimulated with VEGF significantly attracted monocyte migration, which could be abolished by CXCL1 B/N Ab, CXC receptor 2 antagonist, TGF-β, and dexamethasone. In summary, we provide here evidence showing JNK activation for VEGF-induced CXCL1 DNA transcription and PI-3K pathway for extracellular CXCL1 release in human carcinoma epithelial cells. The released CXCL1 was functionally linked to recruiting monocytes into lung cancer cell microenvironment.

## 1. Introduction

CXCL1, also known as growth-related oncogene protein-α (GRO-α) or melanoma growth stimulatory activity factor (MGSA), is a polypeptide which was initially isolated from Hs294 human melanoma cells. CXCL1 is one of the members of chemokines (chemotactic cytokines), which are small heparin-binding proteins that generally direct the movement of circulating leukocytes to sites of inflammation or injury [1]. CXC chemokines (designated ELR^+^), such as CXCL1 and CXCL8, bind the neutrophil receptors CXCR1 and CXCR2 to each other [2]. The ELR^+^ chemokines are primarily chemotactic for endothelial cells and neutrophils. These chemokines are potent promoters of angiogenesis, as the recruited neutrophils are known to synthesize and store angiogenic molecules like vascular endothelial growth factors (VEGFs) [3,4].

VEGF represents a family of homodimeric glycoproteins which are critical for the embryonic development of the blood vascular system (vasculogenesis), lymphatic system (lymphangiogenesis) and in the formation of new blood vessels from pre-existing vessels (angiogenesis) in physiological and pathological conditions [5]. VEGF binds to three different but structure-related tyrosine kinase receptors, including VEGF receptor (VEGFR)-1 (also named as Flt-1), VEGFR-2 (also named as KDR), and VEGFR-3. VEGF-A binds to both VEGFR-1 and VEGFR-2, whilst VEGF-B binds exclusively to VEGFR-1. VEGF-C and VEGF-D are initially expressed as pro-peptides that bind the VEGFR-3 [5]. In addition to VEGFR, VEGF has also been shown to interact with semaphorin receptors (neuropilin-1 and neuropilin-2) [6] and heparan-sulfate proteoglycans [7]. It is now known that VEGFR-1, VEGFR-2, and VEGFR-3 are critical for development of haematopoietic cells, vascular endothelial cells, and lymphatic endothelial cells, respectively. It was reported that in lung cancer patients high expression of VEGF correlates with metastasis. In addition, VEGF secreted by human A549 lung carcinoma cells facilitates tumor metastasis in a murine model [8]. A systematic review of published studies indicates that VEGF overexpression is associated with a poor prognosis in both non-small cell lung cancer (NSCLC) and small cell lung cancers [9]. Some reports have shown that VEGF is induced after irradiation both *in vitro* and *in vivo* in Lewis lung carcinomas (LLC) [10].

In human airway epithelium and bronchoalveolar macrophages, monocyte chemoattractant protein-1 (MCP-1) and CXCL1 were constitutively expressed and upregulated by TNF-α but not by lipopolysaccharide (LPS) [11]. In pathological conditions, various cancer and/or cancer cells express different chemokines and chemokine receptor that modulate leukocyte infiltration within tumor microenvironment, tumor growth and metastasis. For example, CXCL1 has been reported to be expressed in melanoma, breast, colon and ovarian cancer [3]. Non-small cell lung cancer (NSCLC) biopsy specimens have high intratumoral concentrations of CXCR2 ligands (CXCL1, CXCL5, and CXCL8) and type 2 cytokines interleukin-4 (IL-4), IL-5, IL-10, and IL-13 [12,13]. It has also been reported that IL-17 augments the secretion of an array of angiogenic CXC chemokines, including CXCL1, CXCL5, CXCL6, and CXCL8 by three different non-small cell lung cancer cell lines [14]. Recently, CXCL1 was shown to play a pivotal role in thrombin-induced angiogenesis [15].

Considering the significance of CXCL1 in human airway epithelium and in pathological processes such as chronic inflammation and lung cancer, in this study we screened several proinflammatory mediators and growth factors in inducing CXCL1 release in human A549 lung carcinoma epithelial cells. We found a marked enhancing effect by VEGF. Therefore, the effects on CXCL1 release in A549 cells by VEGF were further investigated. We showed that VEGF induced CXCL1 expression through a transcriptional regulation in A549 cells. The possible underlying mechanisms were determined, which showed that VEGF regulated CXCL1 production through JNK- and PI-3K-dependent pathways.

## 2. Results

### 2.1. VEGF Markedly Induces CXCL1 Release in A549 Lung Epithelial Cells

**Figure 1:**
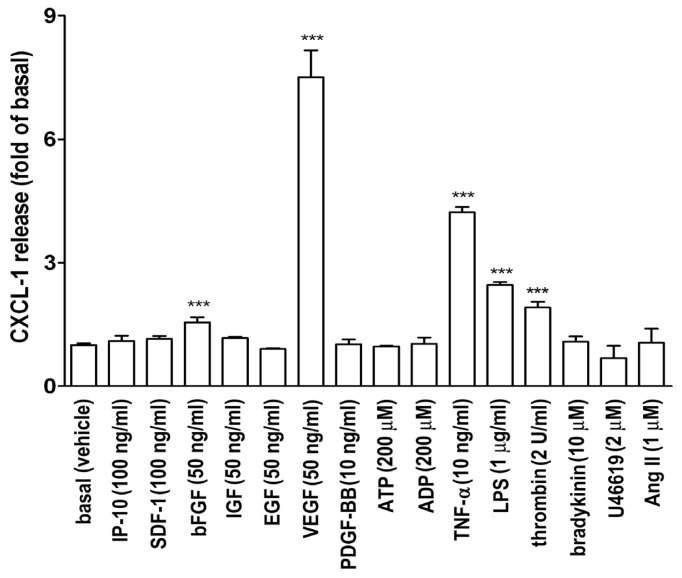
Effect of various mediators on CXCL1 release in A549 epithelial cells. A549 cells were treated with the indicated mediators for 16 h. CXCL1 release in culture medium was measured by ELISA (*n* = 3–4). ***p < 0.001 as compared with vehicle treatment only (basal).

To investigate which proinflammatory cytokines or growth factors affected CXCL1 release in A549 lung epithelial cells, an ELISA for measuring CXCL1 in A549 culture medium was performed. Figure 1 shows that bFGF, VEGF, tumor necrosis factor-α (TNF-α), lipopolysaccharide (LPS), and thrombin induced an increase in CXCL1 release in A549 cell culture medium. Other mediators did not show any significant increase in CXCL1 release. Since VEGF markedly enhanced CXCL1 release, its effect and action mechanism were investigated in this study.

**Figure 2:**
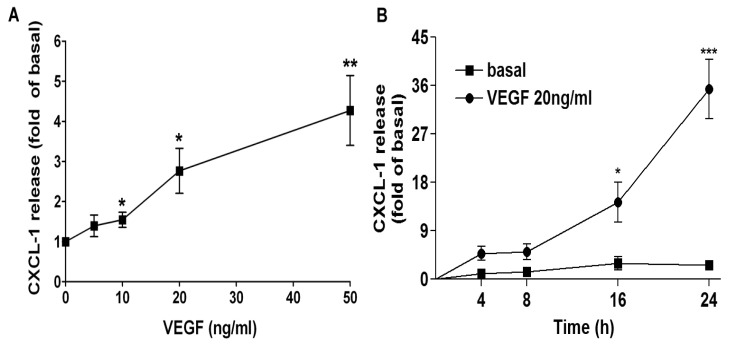
Concentration- and time-dependent effects on VEGF-induced CXCL1 release in A549 cells. A549 cells were treated with (**A**) the indicated concentrations of VEGF for 16 h or (**B**) PBS (basal) or vascular endothelial growth factor (VEGF) for the indicated time intervals. The culture media were collected and analyzed by ELISA. Data were mean ± SEM and expressed as fold of basal (*n* = 4–6). *p < 0.05, **p < 0.01, and ***p < 0.001 *versus* basal level.

Next, we examined the concentration- and time-effect of VEGF on CXCL1 release in A549 lung epithelial cells. As shown in Figure 2, VEGF concentration-dependently increased CXCL1 release, 10 ng/mL of VEGF was sufficient to significantly induce CXCL1 release and 20 ng/mL of VEGF nearly reached to plateau. Moreover, VEGF increased CXCL1 release in a time-dependent manner, a slight increase was observed at a short-term incubation and an apparent increase was found at 16-h treatment.

### 2.2. VEGF Transcriptionally Regulates CXCL1 Expression in A549 Lung Epithelial Cells

**Figure 3:**
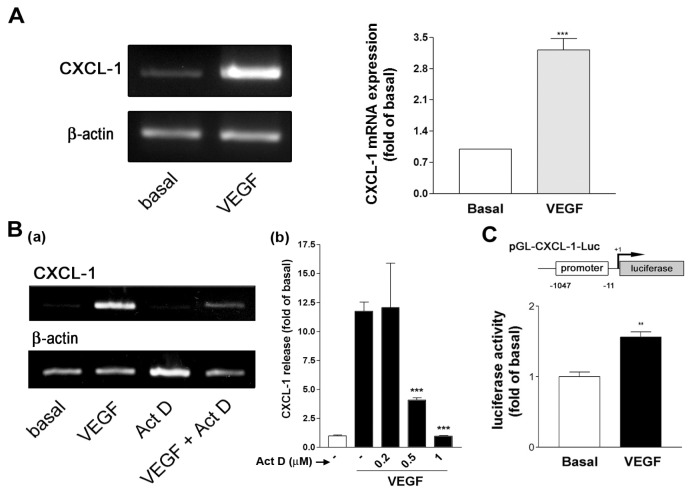
VEGF transcriptionally regulates CXCL1 expression in A549 cells. (**A**) Effect of VEGF on CXCL1 mRNA expression. A549 cells were treated with VEGF (20 ng/mL) for 6 h. At the end of incubation, cells were collected and total RNA was analyzed by RT-PCR. The PCR products for CXCL1 and β-actin were indicated. Data from similar experiments were quantified by densitometry (*n* = 3); (**B**) Effect of transcription inhibitor on VEGF-induced (**a**) CXCL1 mRNA expression and (**b**) CXCL1 release. A549 cells were pretreated with actinomycin D (Act D, 1 μM) or the indicated concentrations of Act D for 30 min and followed by the addition of VEGF (20 ng/mL) for (**a**) 4 h or (**b**) 16 h. CXCL1 mRNA expression was analyzed by RT-PCR (*n* = 3) and CXCL1 release was by ELISA; (**C**) Effect of VEGF on CXCL1 promoter reporter luciferase activity. Cells were transfected with CXCL1 promoter reporter and stimulated with vehicle or VEGF (20 ng/mL). Data were luciferase intensity ratio to β-gal activity and were normalized to basal (vehicle treatment) (*n* = 3). **p < 0.01 and ***p < 0.001 *versus* basal level or VEGF control.

To further examine whether VEGF induced CXCL1 mRNA expression, A549 cells were treated with VEGF and CXCL1 and β-actin mRNA expression was evaluated by RT-PCR. As shown in Figure 3A, CXCL1 mRNA was upregulated by VEGF, whereas β-actin mRNA expression was not affected. This suggested that VEGF might affect CXCL1 expression through a transcriptional regulation. To confirm this hypothesis, a gene transcription inhibitor actinomycin D (Act. D) was used to examine whether it affected VEGF-induced CXCL1 release. It was shown that Act. D reduced VEGF-induced CXCL1 mRNA expression [Figure 3B(a)] and CXCL1 release in a concentration-dependent manner [Figure 3B(b)]. In addition, an incubation of cells transfected with a CXCL1 promoter region-constructed luciferase reporter with VEGF resulted in an enhanced luciferase activity in A549 cells (Figure 3C), suggesting that CXCL1 DNA transcription was involved in VEGF-induced CXCL1 release.

### 2.3. Effect of Signaling Inhibitors on VEGF-Induced CXCL1 Expression in A549 Cells

**Figure 4:**
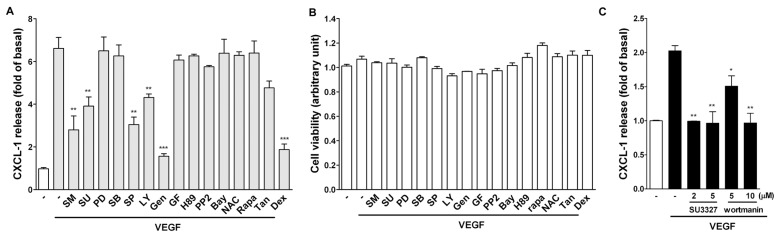
Effect of signaling inhibitors on CXCL1 release in A549 cells. A549 cells were pretreated with various inhibitors (SM and SU: 5 μM; PD, SB, SP and LY: 10 μM; gen: 10 μg/mL; GF and PP2: 2 μM; H-89 and Bay: 5 μM; NAC: 1 mM; rapamycin (rapa, 1 μg/mL); Tanshinone IIA (Tan, 10 μM); dexamethasone (DEX, 100 nM)) for 0.5 h and followed by PBS (basal) or VEGF (20 ng/mL) for 16 h. The CXCL1 in culture media was analyzed by ELISA, and the remaining cells were examined by MTT assay. Data were mean ± SEM (*n* = 3–5). **p < 0.01 and ***p < 0.001 *versus* VEGF control.

To investigate the possible signaling pathways involved in the induction of CXCL1 by VEGF, signaling inhibitors targeting MAPKs, PI-3K, protein kinases, NF-κB signaling pathway, and DNA transcription were used. Among these inhibitors, it was found that the CXCL1 release by VEGF was significantly affected by the following inhibitors, including the VEGFR antagonists (Sunitinib and SU5416), JNK inhibitor (SP600125; SP), PI-3K inhibitor (LY294002; LY), and tyrosine kinase inhibitor (genistein; gen) (Figure 4A). Moreover, it was found that the steroid dexamethasone (DEX, 100 nM) markedly inhibited VEGF-induced CXCL1 release. The inhibition was not due to decrease of cell viability because these inhibitors did not affect cell viability (Figure 4B). To confirm JNK and PI-3K in VEGF-induced CXCL1 release, other inhibitors for JNK and PI-3K was used. As shown in Figure 4C, SU3327 (a JNK inhibitor) and wortmanin (a PI-3K inhibitor) also inhibited VEGF-induced CXCL1 release.

**Figure 5:**
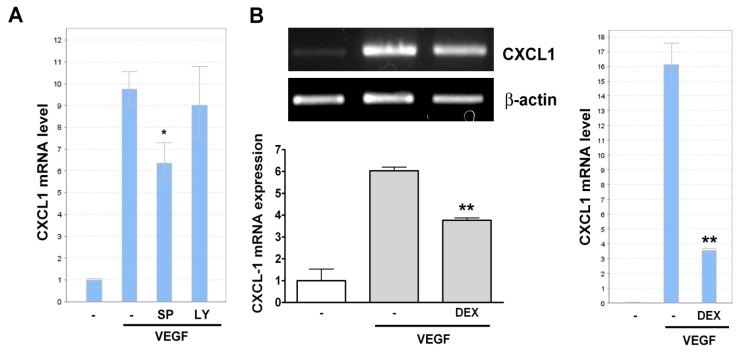
Effect of signaling inhibitors on CXCL1 mRNA level in A549 cells. A549 lung cancer cells were pretreated with (**A**) SP600125 (SP, 10 μM) and LY294002 (LY, 10 μM) or (**B**) dexamethasone (DEX, 100 nM) for 0.5 h and followed by stimulation with 20 ng/mL of VEGF for 4 h. Total RNA were extracted by Trizol reagent and analyzed by RT-PCR or real-time PCR. Data were mean ± SEM (*n* = 3). *p < 0.05 and **p < 0.01 *versus* VEGF control.

We next examined whether SP and LY had a similar effect on VEGF-induced CXCL1 mRNA expression. Surprisingly, the real-time PCR analysis indicated that only SP reduced VEGF-induced CXCL1 mRNA expression, whereas LY had no such inhibitory effect (Figure 5A). The RT- and real-time PCR analysis also demonstrated that dexamethasone reduced VEGF-induced CXCL1 mRNA expression (Figure 5B). Taken together, these results suggested that VEGF-induced JNK activation mediated CXCL1 mRNA transcription, whereas PI-3K pathway might be related to extracellular CXCL1 release. In addition, dexamethasone compromised VEGF-induced CXCL1 release through a transcriptional regulation.

### 2.4. VEGF Directly Induces JNK and PI-3K Activation

**Figure 6:**
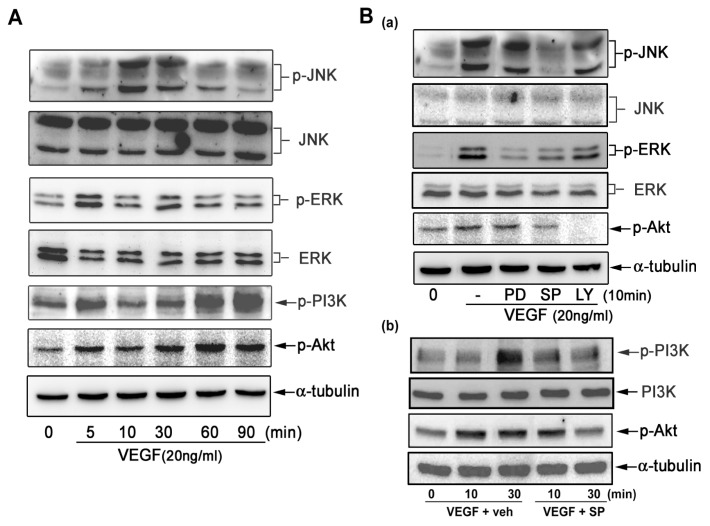
VEGF induces MAPKs, PI3K, and Akt activation in A549 cells. A549 lung cancer cells were treated with (**A**) VEGF for indicated time intervals or (**B**) various signaling inhibitors (10 μM for each) for 30 min and followed by VEGF stimulation. After incubation, cell lysates were analyzed Western blotting. A representative blot was shown (**upper panel**) and similar results were quantified by densitometry (**lower panel**) (*n* = 3–4).

As JNK and PI-3K inhibitors reduced VEGF-induced CXCL1 release, we next examined whether VEGF could directly activate related signaling pathways in A549 cells. Figure 6A shows that VEGF markedly activated JNK and PI-3K in A549 cells and slightly activated ERK1/2. It was found that VEGF-induced JNK, PI-3K, and Akt activation was in a two-phase fashion, which was activated at 5–30 min but returned to basal level and followed by an increase about at 90 min. Next we determined the activation context of JNK and PI-3K in VEGF-induced CXCL1 release. The Western blot analysis demonstrated that the JNK inhibitor (SP) not only inhibited JNK activation but also inhibited PI-3K and Akt activation [Figure 6B(a,b)]. On the contrary, the PI-3K inhibitor (LY) inhibited PI-3K and Akt activation but had no effect on JNK activation [Figure 6B(a)]. This finding explained the kinase activation context in A549 cells in response to VEGF.

### 2.5. Role of Released CXCL1 by A549 Cells in Attracting Monocyte Migration

**Figure 7:**
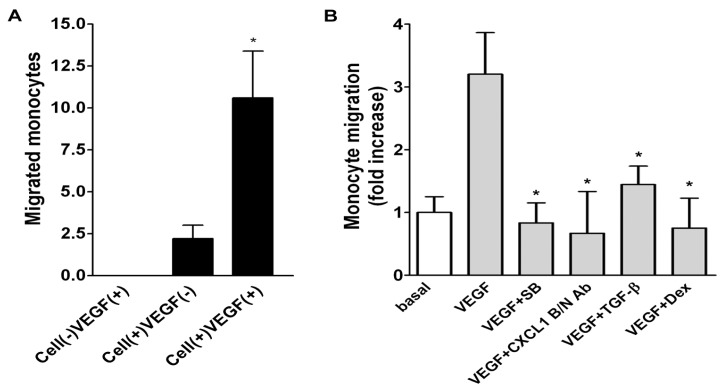
Role of released CXCL1 in monocyte migration. The transwell insert precoated with gelatin were seeded with monocytes. (**A** and **B**) The upper chamber (insert) was assembled with the lower chamber seeded with/without A549 lung epithelial cells in the presence of VEGF (20 ng/mL) and the indicated agents (SB225002 (SB, 0.5 μM), CXCL1 B/N Ab (10 μg/mL), TGF-β (5 ng/mL), or DEX (100 nM)). After incubation for 16 h, the migrated monocytes were fixed and counted by microscopy (*n* = 2–3). Cell (+/−) and VEGF (+/−) in (**A**) indicate presence/absence of the seeded A549 and VEGF in the lower chamber, respectively. *p < 0.05 *versus* control.

To evaluate the functional role of CXCL1 secretion by A549 cells in recruiting monocyte migration, a modified Boyden chamber transmigration system was used. The lower chamber was seeded with/without monolayered A549 cells, which assembled with the upper chamber added with U937 monocytes to form a coculture system. In the absence of A549 cells but presence of VEGF, there were no migrated monocytes, indicating that VEGF alone was not sufficient to cause monocyte migration. However, a slight increase of migrated monocytes was observed in the presence of seeded A549 cells in the lower chamber and a robust increase of monocytes in the presence of A549 cells stimulated with VEGF (Figure 7A), suggesting VEGF working as a potent inducer for A549 cells to secrete a mediator attracting monocyte migration. We next examined whether SB225002 (SB, a selective antagonist for CXC receptor 2 (CXCR2)) [16], affected A549 cells-induced monocyte migration. As shown in Figure 7B, SB225002 completely inhibited A549 cells/VEGF-dependent monocyte migration (left panel). Moreover, the monocyte migration was reduced by CXCL1 blocking/neutralizing Ab (B/N Ab), dexamethasone, and TGF-β (right panel).

### 2.6. Effect of TGF-β on VEGF-Induced CXCL1 Expression

**Figure 8:**
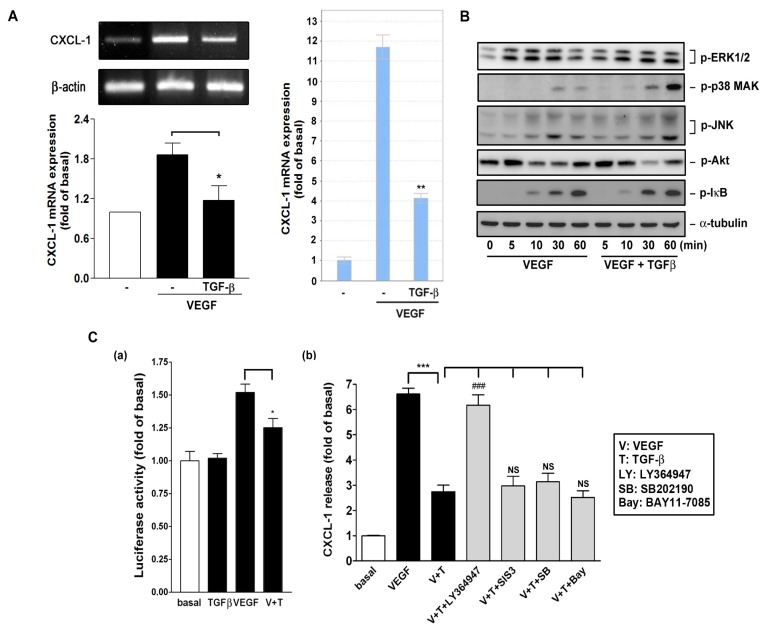
Effect of TGF-β on CXCL1 expression and release in A549 cells. (**A**) Effect of TGF-β on VEGF-induced CXCL1 mRNA expression. A549 cells were treated with VEGF (20 ng/mL) for 6 h. At the end of incubation, cells were collected and total RNA was analyzed by RT-PCR and real-time PCR. Data from similar experiments were quantified (*n* = 3); (**B**) Effect of TGF-β on VEGF signaling. A549 lung epithelial cells were treated with VEGF in the absence or presence of TGF-β (10 ng/mL) for the indicated time. Cell lysates were analyzed by Western blotting (*n* = 3); (**C**) Effect of TGF-β on VEGF-induced (**a**) CXCL1 luciferase reporter activity and (**b**) CXCL1 release. Cells were treated with VEGF (20 ng/mL) and TGF-β (10 ng/mL) in the absence or presence of the indicated inhibitors (LY: 1 μM, SIS3: 10 μM, SB: 10 μM, Bay: 10 μM). The luciferase activity was measured by luminometry and CXCL1 release was determined by ELISA (*n* = 3–4). *p < 0.05, **p < 0.01, and ***p < 0.001 *versus* VEGF control. ^###^*p* < 0.001 *versus* VEGF + TGF-β (*n* = 3).

Alterations in TGF-β signaling are linked to a variety of human diseases, including cancer and inflammation. Disruption of TGF-β homeostasis occurs in several human cancers such as lung cancer [17–19]. TGF-β has a vital role in suppressing the activation and proliferation of inflammatory cells [20]. TGF-β is important in suppressing primary tumorigenesis in many tissue types [21]. However, many human cancers, including lung cancer, often overexpress TGF-β and TGF-β enhances the invasiveness and metastatic potential in certain late-stage tumors [22]. In Figure 7B, we have shown that TGF-β functionally affected A549 cells-induced monocyte migration. Therefore, we tested if TGF-β affected VEGF-induced CXCL1 expression. As shown in Figure 8A, TGF-β significantly inhibited VEGF-induced CXCL1 mRNA expression, as determined by RT- and quantitative real-time PCR analysis. However, TGF-β did not interfere with VEGF signaling such as JNK and Akt pathways required for CXCL1 release (Figure 8B). Figure 8C shows that TGF-β affected VEGF-induced luciferase activity (panel a), suggesting that TGF-β affected CXCL1 transcription by VEGF. In addition, Figure 8C(b) shows that the inhibition of CXCL1 release by TGF-β could be reversed by the antagonist LY364947 (LY) for TGF-β type I receptor, which is known to mediate its signaling through heterodimering with TGF-β type II receptor [23]. However, it could not be reversed by SIS3 (a Smad3 inhibitor), SB202190 (a p38 MAPK inhibitor), and BAY11-7085 (a NFκB signaling inhibitor).

## 3. Discussion

Some of the chemokines and cytokines have been found to be regulated in the *in vitro* model (CCL2, VEGF, CCL5, CXCL1, CXCL2, and CCL11) are also highly expressed in lung tumors in mice and humans [13,24,25]. In this study we found that VEGF, bFGF, TNF-α, LPS and thrombin could induce CXCL1 release in A549 lung epithelial carcinoma cells (Figure 1). Among these stimulators, VEGF induced a robust increase in CXCL1 release in A549 cells. Therefore, the effect and mechanism of action of VEGF was further investigated. The inductory effects by VEGF were through a transcriptional regulation and possibly a cellular secretory process, which were resulted from JNK and PI-3K related pathways, respectively. More importantly, a modified Boyden chamber coculture system demonstrated an ability of secreted CXCL1 in attracting monocyte migration (Figure 7), suggesting that the increased CXCL1 was functionally linked to attracting of monocyte migration toward to lung A549 cells in response to VEGF.

It has been shown that NF-κB mediates IL-1/TNF-α induction of CXCL1 in human fibroblasts [26] and protein kinase D (also named as PKC-μ) mediates VEGF-induced proinflammatory cytokines such as CXCL1, CXCL8 and IL-6 in human vascular endothelial cells [27]. In this study, however, a general PKC inhibitor (GF109203X), PKA inhibitor (H-89), and NF-κB signaling inhibitor (Bay) did not affect VEGF-induced CXCL1 release (Figure 4), suggesting the process did not involve PKA, PKC, PKD and NF-κB signaling pathways. VEGF induces CXCL1 expression through a transcriptional regulation, which is evidenced by the following findings. First, VEGF enhanced CXCL1 mRNA transcription and a gene transcription inhibitor-actinomycin D could attenuate VEGF-induced CXCL1 mRNA expression and protein release (Figure 3A,B). Secondly, the luciferase reporter analysis indicated that VEGF could increase luciferase activity in A549 cells transfected with the CXCL1 reporter construct (Figure 3C).

VEGF-A binds to VEGFR1 and VEGFR2. VEGFR1 tyrosine kinase activity is only weakly induced by its ligands [28]. A range of signaling molecules associate with VEGFR1 phosphorylation sites *in vitro*, including phospholipase Cγ (PLCγ), PI-3K, ERK1/2 and *etc.* However, VEGFR1 has been shown to regulate endothelial cells (ECs) via cross-talk with VEGFR-2. VEGFR-2 is the principal mediator of several physiological and pathological effects of VEGF-A on ECs. The intracellular signaling pathways mediating these effects downstream of VEGFR-2 activation include PLCγ, p38 MAPK, PI-3K, ERK1/2 and *etc.* [5]. Human A549 cell has been shown to express VEGFR2 and its activation can be inhibited by a clinically used tyrosine kinase inhibitor [29]. In this study, VEGF-induced CXCL1 production was significantly inhibited by the VEGF receptor inhibitors (SM and SU), JNK inhibitor (SP), PI-3K inhibitor (LY), tyrosine kinase inhibitor (gen), and the steroid dexamethasone but not by other inhibitors (Figure 4). However, in contrast to their marked inhibitory effect on CXCL1 release, only the JNK inhibitor but not PI-3K inhibitor reduced VEGF-induced CXCL1 mRNA expression (Figure 5A). Therefore, it is suggested that VEGF activates VEGFR and induces CXCL1 release through two differential pathways, one affects CXCL1 transcription through JNK activation and the other affects cellular CXCL1 secretion through PI-3K activation. This was supported by the observations that VEGF-induced CXCL1 release could also be reduced by other JNK (SU3327) and PI-3K inhibitor (wortmanin) (Figure 4C) and VEGF directly and markedly activated JNK, PI-3K and Akt in A549 epithelial cells (Figure 6). It has been shown that JNK, when active as a dimer, can translocate to the nucleus and regulate transcription through its effects on AP-1 transcription factors [30,31]. However, in this study the downstream transcription factor responsible for JNK-mediated *CXCL1* DNA transcription needs to be further investigated as Tanshinone IIA (an AP-1 inhibitor) did not significantly affect VEGF-induced CXCL1 release (Figure 4). It is interesting that VEGF affects CXCL1 release through two distinct pathways in A549 epithelial cells, which is quite different from that in human vascular ECs through a PKD-dependent pathway [27]. To our knowledge, little is known about the secretion pathways responsible for chemokine release. Some studies showed that the storage and release of IL-8 (CXCL8) from secretory vesicles are loaded by endocytosis during late stages of neutrophil development in the bone marrow but is still controversial [32,33]. A detailed understanding of how VEGF regulates CXCL1 release merits a further study.

Another finding from the present study is that dexamethasone and TGF-β regulated VEGF-induced CXCL1 release (Figures 4,8) and affected A549 cells/VEGF-induced monocyte migration (Figure 7B). A previous study has shown that dexamethasone inhibits TNF-α-induced CXCL1 secretion in human tracheal smooth muscle cells (HTSMCs) through induction of MAPK phosphatase-1 (MKP-1) expression and thus dephosphorylates phosphorylated JNK, leading inactivation of JNK required for CXCL1 transcription [34]. As dexamethasone also compromised VEGF-induced CXCL1 mRNA expression (Figure 5B), it possibly acted on A549 cells in a similar way to HTSMCs. Interestingly, dexamethasone failed to inhibit TNF-α-induced CXCL1 secretion in human vascular ECs (our unpublished data), indicating a differential effect of dexamethasone on certain cell types. It has been shown that TGF-β inhibited TNF-α-induced CXCL1 release in human ECs [35] and TGF-β regulated suppression of inflammatory genes such as CXCL1 and CXCL5 in mammary carcinoma cells [36]. In this study, we demonstrated that TGF-β affected VEGF-induced CXCL1 mRNA level and luciferase reporter activity, suggesting it might interfere with VEGF-induced CXCL1 release through a transcriptional mechanism. As reported by others, all TGF ligands transmit biological information to cells by binding to type I and type II receptors that form heterotetrameric complexes in the presence of the dimeric ligand, which interacts with other proteins and subsequently leads to Smad homo- and hetero-oligomerization and mediates the transactivation potential of nuclear Smad complexes [37]. In addition to the activation of Smad-dependent cascades, TGF-β can also signal in a noncanonical fashion, *i.e.*, MAPKs pathways [38,39]. We showed that TGF-βRI antagonist completely reversed TGF-β inhibition but the Smad3, p38 MAPK and NF-κB signaling inhibitors did not, suggesting involvement of activation of TGFR1 but not of downstream Smad3, p38 MAPK and NF-κB during this process. Because TGF-β did not affect cytosolic signaling pathways by VEGF but it reduced CXCL1 luciferase reporter activity by VEGF (Figure 8B,C), it is possible that TGF-β affects VEGF-induced CXCL1 promoter activity. TGF-β has been suggested to be as a tumor suppressor or promoter [40]. However, in lung cancer, overexpression of TGF-β is associated with better prognosis in 5-year patient survival [41]. Although its inhibitory mechanism on VEGF-induced CXCL1 release remains to be determined, our results reveal that TGF-β downregulates CXCL1 chemokine expression and reduces leukocyte migration. These explain that TGF-β may have anti-inflammatory activity, reducing leukocyte infiltration in tumor microenvironment and interfering with tumorigenesis.

## 4. Experimental Section

### 4.1. Materials

Thrombin, bradykinin, PD98059, SB202190, SP600125, 3-[4,5-dimethylthiazol-2-yl]-2,5-diphenyltetrazolium bromide (MTT), wortmanin, and actinomycin D were purchased from Sigma Chemical Co. (St. Louis, MO, USA). SU3327 was from Tocris Bioscience (Bristol, UK). Human recombinant VEGF-A was purchased from Prospec Biotech (Rehovot, Israel). Human EGF, IGF, and bFGF were from Invitrogen Life Technologies (Carlsbad, CA, USA). The antibody (Ab) raised against phospho-ERK1/2 was from Santa Cruz Biotechnology (Santa Cruz, CA, USA). The Abs raised against total p38 MAPK, phospho-p38 MAPK, and phospho-JNK were from Cell Signaling Technology, Inc. (Danvers, MA, USA). Human IP-10, SDF-1, PDGF, TNF-α, and the Abs for total p38, ERK1/2, and CXCL1 blocking/neutralizing Ab (B/N Ab) were from R & D systems, Inc. (Minneapolis, MN, USA). ATP and ADP were purchased from Affymetrix USB Products (Santa Clara, CA, USA). U46619 was from Enzo Life Sciences, Inc. (Farmingdale, NY, USA). Sunitinib malate was from Biovision (Milpitas, CA, USA) and SU5416 was from Cayman Chemical Co. (Ann Arbor, MI, USA). SIS3 and α-tubulin Ab were purchased from Calbiochem EMD Bioscience Inc. (San Diego, CA, USA).

### 4.2. Cell Culture

A549 cells, a human pulmonary epithelial carcinoma cell line with type II alveolar epithelial cell differentiation, were from Food Industry Research and Development Institute (Hsinchu, Taiwan). The cells were cultured in DMEM/Ham’s F-12 nutrient mixture containing 10% FBS (fetal bovine serum), penicillin (100 units/mL), streptomycin (100 μg/mL) and fungizone (250 ng/mL) (Invitrogen Life Technologies, Carlsbad, CA, USA). U937 monocytes were also from Food Industry Research and Development Institute and cultured in RPMI 1640 medium with 2 mM l-glutamine, 1.5 g/L sodium bicarbonate, 4.5 g/L glucose, 10 mM HEPES, and 1.0 mM sodium pyruvate and 10% FBS.

### 4.3. Measurement of Secreted CXCL1 in Culture Medium by ELISA

Secreted CXCL1 in culture medium was determined by human CXCL1 ELISA Development kit (R & D Systems, Inc., Minneapolis, MN, USA) according to the manufacturer’s protocol. Briefly, A549 cells were treated with vehicle or stimulators. The culture media were collected and centrifuged and CXCL1 release in culture medium was measured. The product of this enzymatic reaction was yellowish color and absorbs strongly at 412 nm. The intensity of this color is proportional to the amount of CXCL1 present in the well after the incubation. The CXCL1 concentrations in A549 cell culture medium were calculated from the standard curve.

### 4.4. Cell Viability Assay

Cell viability was assayed as previously described [42]. Briefly, the cells were incubated with 0.5 mg/mL MTT for 2 h at 37 °C. Formazan crystals resulting from MTT reduction were dissolved by adding DMSO. The absorbance of the supernatant was then measured spectrophotometrically in an ELISA reader at 550 nm.

### 4.5. Cell Lysate Preparation and Western Blot Analysis

Cell lysate was prepared as previously described [43]. Total proteins were separated by electrophoresis on SDS-polyacrylamide gels, electroblotted onto PVDF membranes, and then probed using a primary mAb. Immunoblots were detected by enhanced chemiluminescence reagent (Perkin-Elmer, Waltham, MA, USA). For some experiments, membranes were stripped with a striping buffer (62.5 mM Tris-HCl, pH 6.7, 2% SDS and 100 mM β-mercaptoethanol), washed, and reprobed with Abs for the levels of α-tubulin or the corresponding total proteins and developed as described above.

### 4.6. Reverse Transcription-Polymerase Chain Reaction (RT-PCR) and Real-Time PCR Analysis of CXCL1 mRNA Expression

Oligonucleotide PCR primers targeting to human CXCL1 and β-actin were synthesized. The forward and reverse primers for CXCL1 were 5′-GCCCAAACCGAAGTCATAGCC-3′ and the forward and reverse primers for β-actin were 5′-ATCATGTTTGAGACCTTCAA-3′ and 5′-CATCTCTTGCTCGAAGTCCA-3′, respectively. Total RNA of A549 cells was extracted by Trizol reagents (Invitrogen Technologies) and reverse transcription reaction was performed by using Superscript III First-Strand Synthesis System (Invitrogen Technologies). Briefly, aliquots of 1–2 μg total RNA were incubated with random hexaprimers for 10 min at 65 °C and chilled on ice shortly. After primer annealing, RNA was reverse transcribed by the reverse transcriptase. Reactions were stopped and RNase H was added to remove RNA. Aliquots of transcribed cDNA were subjected to PCR in 25 μL of reaction mixture containing reaction buffer, dNTP, primers, and *Taq* DNA polymerase (Genet Bio, Daejeon, Korea). PCR was performed with a hot start at 94 °C for 5 min and then with 30 cycles of denaturation at 94 °C for 1 min, annealing at 56 °C for 1 min, and elongation at 72 °C for 1.5 min on the ABI 7200 Thermal Cycler (Applied Biosystems, Foster City, CA, USA). The amplification products were then analyzed by gel electrophoresis in 2% agarose. For some experiments, CXCL1 mRNA level was analyzed by real-time PCR with the TaqMan gene expression assay system (Life Technologies, Applied Biosystems, Grand Island, NY, USA), using primers/probe sets Hs.708652 for human CXCL1 and Hs.520640 for human β-actin (as a control). PCRs were performed using a 7500 Real-Time PCR System (Life Technologies, Applied Biosystems, Grand Island, NY, USA). Relative gene expression was determined by the ΔΔCt method, where Ct was the threshold cycle. All experiments were performed in duplicate or triplicate.

### 4.7. CXCL1 Reporter Construct, Transfection, and Luciferase Assay

The wild-type CXCL1 promoter fragment spanning nucleotides −1047 to −11 of the CXCL1 promoter cloned into pXP2 luciferase reporter plasmid was cloned. Briefly, the region was amplified from genomic DNA of A549 cells using the primers with linkers and restriction enzyme sites for cloning to the pGL3-luciferase reporter plasmid. The primers were 5′-GTGGAAGGTGCTT GCACACCAGG-3′ (forward) and 5′-GGAGCAGCAGTGCCACTCGCAGG-3′ (reverse). The accuracy of CXCL1 sequence was confirmed by DNA sequencing. Cells at approximately 80% confluence in 6-well culture plate (Corning Incorporated Life Sciences, Tewksbury, MA, USA) were transfected with 0.75 μg of total DNA, using PolyJet™ *in vitro* DNA Transfection Reagent (SignaGen Lab, Rockville, MD, USA) for 18 h in medium according to the manufacturer’s protocol. All DNAs were prepared using endotoxin-free plasmid preparation kits (Qiagen, Hamburg, Germany). All transient transfections included 0.375 μg of CXCL1 reporter construct and pSV-β-galactosidase control vector (Promega, Madison, WI, USA). Following transfection, cells were washed once with endotoxin-free medium and then allowed to grow for 16 h in complete medium containing antibiotics. CXCL1 reporter firefly luciferase values were obtained by analyzing 1 mL of purified cell extract according to standard instructions provided by the Luciferase Kit (Promega, Madison, WI, USA) in a Wallac Victor 3 1420 multilabel counter (Perkin Elmer, Turku, Finland).

### 4.8. Measurement of A549 Cells-Induced Monocyte Migration

Monocyte migration assay was performed using a modified Boyden chamber model (Transwell apparatus, 5.0-μm pore size, Falcon). The lower chamber was seeded with/without A549 cells. After 90% of confluency, cells were filled with serum-free or VEGF-containing medium in the presence of vehicle, CXCL1 B/N Ab, CXCR2 inhibitor, TGF-β, or dexamethasone. The lower face of polycarbonate filters (Transwell insert) were coated with gelatin (20 μg/mL) for 30 min in the laminar flow hood. The upper chamber was loaded with human U937 monocytes (2.5 × 10^5^ cells/mL) and then assembled with the lower chamber. The coculture system was allowed to incubate at 37 °C for 16 h. All nonmigrant monocytes were removed from the upper face of the Transwell membrane with a cotton swab and migrated monocytes were fixed and stained with 0.5% toluidene blue in 4% paraformaldehyde. Migration was quantified by counting the number of stained cells per × 100 field (high power field, HPF) under a phase-contrast microscope (Nikon Eclipse Ti-S, Shinagawa-ku, Tokyo, Japan) and photographed.

### 4.9. Statistical Analysis

Data were expressed as mean ± standard error of the mean (SEM). The means of two groups of data were compared using the unpaired, two-tailed Student’s *t*-test.

## 5. Conclusions

In conclusion, in the present study we demonstrate that VEGF can induce CXCL1 mRNA and protein expression in A549 carcinoma epithelial cells through VEGFR, JNK and PI-3K-dependent pathway. Our results suggest that JNK is essential for CXCL1 synthesis, whereas PI-3K is for cellular CXCL1 release. The induction of CXCL1 release by VEGF in A549 cells functionally leads to the recruitment of monocytes toward themselves in the microenvironment. Lung cancer and/or cancer cells express different chemokines that chemokine receptor that modulate leukocyte infiltration within tumor microenvironment. Our results suggest the contribution of VEGF and elucidate its possible mechanism in causing CXCL1 release.
